# Mucin 5B Promoter Polymorphism Is Associated with Susceptibility to Interstitial Lung Diseases in Chinese Males

**DOI:** 10.1371/journal.pone.0104919

**Published:** 2014-08-14

**Authors:** Chunli Wang, Yi Zhuang, Wenwen Guo, Lili Cao, Huan Zhang, Lizhi Xu, Yimei Fan, Deping Zhang, Yaping Wang

**Affiliations:** 1 Department of Medical Genetics, Nanjing University School of Medicine, Nanjing, China; 2 Jiangsu Key Laboratory of Molecular Medicine, Nanjing University School of Medicine, Nanjing, China; 3 Department of Respirology, Medical School affiliated Drum Tower Hospital, Nanjing University, Nanjing, China; Helmholtz Zentrum München/Ludwig-Maximilians-University Munich, Germany

## Abstract

The variation of G>T in the *MUC5B* promoter (rs35705950) has been associated with idiopathic pulmonary fibrosis (IPF) and familial interstitial pneumonia (FIP) in Caucasians, but no information is available regarding this variant in the Chinese population. We recruited 405 patients with interstitial lung diseases (ILD), including 165 IPF patients and 2043 healthy controls, for genotyping the *MUC5B* gene in the Chinese population. One hundred three patients with pneumonia and 360 patients with autoimmune diseases (ADs) were recruited as disease controls. Our results indicated that the prevalence of the minor allele (T) of the polymorphism rs35705950 in healthy Chinese subjects was approximately 0.66%, which was lower than that described in the Caucasian population. The frequencies of the T allele were 3.33% and 2.22% in IPF and ILD patients, respectively, and these values were significantly higher than those of healthy controls (*P* = 0.001, OR = 4.332 for IPF, and *P* = 0.002, OR = 2.855 for ILD). A stratified analysis showed that this variant in *MUC5B* associated with the risk for ILD mainly in older male Chinese subjects. No difference was observed between patients with pneumonia, AD patients, and healthy controls.

## Introduction

Interstitial lung diseases (ILD) or diffuse interstitial lung diseases (DILD) are a heterogeneous collection of more than 100 different pulmonary disorders that affect the tissue and spaces surrounding the alveoli,cause irreversible architectural distortion and impair gas exchange [1], [2]. The most common and aggressive idiopathic interstitial pneumonia is idiopathic pulmonary fibrosis (IPF), which represents a chronic, progressive and typically lethal lung disorder of unknown etiology [3]. The incidence of IPF increases with advancing age. It occurs primarily in middle-aged to older adults and peaks in those over 75 years of age. However, the published prevalence of IPF ranges from 0.7 per 100,000 in East Asia to 63.0 per 100,000 in the United States [4], and the prevalence is higher in men than in women, although this difference between genders was not observed in a population-based investigation from Finland [5]. Some potential risk factors, such as cigarette smoking and other environmental exposures, have been described for IPF [6]. The risk for IPF is likely determined by multiple genetic variants and environmental factors [7].

Most cases of IPF are sporadic, but this disorder can occasionally occur in familial form, which is defined as IPF occurring in two or more first-degree relatives within the same family. The telomerase-related genes (TERT and TERC) [8], [9], surfactant proteins C (SPC) and A2 (SPA2) have been identified to be associated with familial IPF [8], [10]. Heterozygous mutations in either TERT or TERC have been found in approximately 18% of familial IPF and in only 1–3% of sporadic IPF patients. In addition, a variety of investigations have been undertaken in an attempt to define the potential genetic susceptibility for sporadic IPF. Many of these studies focused on the polymorphisms of cytokines, growth factors, and the human leukocyte antigen (HLA) group [11]–[13]. However, the genetic variants that had been implicated in IPF account for only a small proportion of the population risk.

Seibold et al. [14] first used linkage and fine mapping to identify a region of interest on the p-terminus of chromosome 11 that included gel-forming mucin genes. The single-nucleotide polymorphism (SNP) rs35705950 is located 3 kb upstream of the *MUC5B* transcription start site on the gene encoding the Mucin 5 subtype B, which is a gel-forming mucin and a major component of mucus in the respiratory tract [15], [16]. The polymorphism of the *MUC5B* gene has a profound effect on the risk of familial interstitial pneumonia and sporadic IPF in the American population [14], [17]. The subjects who were heterozygous or homozygous for the minor allele of this *MUC5B* polymorphism rs35705950 have a significantly increased risk for IPF (OR = 6.8 and 20.8) and for sporadic IPF (OR = 9.0 and 21.8), respectively [14]. The strong association of the *MUC5B* variant with idiopathic pulmonary fibrosis was recently confirmed in other European Caucasian populations, including Italian, French, and British cohorts [18]–[20], and confirmed in two genome-wide association studies [21], [22]. The gene association studies in the Caucasian population failed to uncover any association between this polymorphism variant and lung fibrosis in the context of systemic sclerosis or sarcoidosis [18]–[20].

ILD is commonly encountered in patients with autoimmune connective tissue diseases and can lead to significant morbidity and shortened survival. Lung involvement occurs in a large proportion (approximately 80%) of patients with systemic sclerosis (SSc) [23], [24], is associated with a poorer quality of life, the need for long-term treatment and a worse prognosis. The autoimmune diseases rheumatoid arthritis (RA), Sjögren's syndrome (SS), and systemic lupus erythematosus (SLE) are associated with a high risk for the development of ILD [25]. However, the pulmonary fibrosis-associated *MUC5B* promoter variant does not influence the development of interstitial pneumonia in patients with systemic sclerosis or sarcoidosis [18]–[20].

Clinical data indicated a significantly increased incidence of ILD in the Chinese population. A retrospective analysis of hospitalized cases in ten large hospitals in 2004 showed that the percentage of DILD patients increased from 1.98% to 4.66% between 1990 and 2003 in Chinese patients hospitalized for respiratory diseases [26]. In Asian populations, polymorphisms of the genes encoding for cytokines IL-3 and TGF-β[27], [28] could affect the susceptibility to IPF. HLA-A and HLA-B *immune* gene polymorphisms [12] were suggested to be associated with IPF in the Korean population. To date, no information is available regarding the putative association between *MUC5B* rs35705950 and fibrotic lung disease in the Chinese population. In the present study, we performed cohort analyses to investigate the relationship between the polymorphism variant of *MUC5B* rs35705950 and the risk for ILD in the Chinese population. We found that the frequency of the minor allele of this polymorphism was much lower in the Chinese population than that described in Caucasian populations. However, a strong association was observed between this variant and the risk for ILD in Chinese subjects, especially in older males.

## Materials and Methods

### Ethics statement

The study protocol was reviewed and approved by the ethics committee of the Nanjing University School of Medicine. Written informed consent was obtained from all of the recruited patients and control subjects before any study procedure.

### Subjects

In this study, we consecutively recruited 508 Chinese patients with lung diseases from 2007 to 2013, including 405 ILD patients and 103 pneumonia patients who were attending the clinics of the Drum Tower Hospital Affiliated with the Medical School of Nanjing University. We collected the following clinical data at our institution: age, gender, past medical history, smoking history, occupational exposure history, physical examination findings, laboratory results, and high resolution computed tomography (HRCT) scans. Respiratory specialists diagnosed the recruited ILD patients based on clinical features and HRCT evaluation, and a histopathologic diagnosis was made for the cases with an atypical HRCT image. The presence of a ground glass opacity and/or reticular opacities or honeycomb cysts in a peripheral distribution on chest HRCT scans of patients was defined as ILD, and these patients were categorized into groups according to their clinical features and the 2011 ATS/ERS consensus: IPF (165 cases), exclusion of the known causes of interstitial lung disease, and the autoimmune connective tissue disease-associated ILD (CTD-ILD, 240 cases) [2], [6]. The latter group included 49 CTD-UIP cases whose HRCT showed the usual interstitial pneumonia pattern and 191 CTD-NSIP cases whose HRCT showed a nonspecific interstitial pneumonia pattern [29]–[31]. The diffuse parenchymal lung diseases (DPLDs) consist of disorders of known causes (environmental or drug related), chronic hypersensitivity pneumonia (CHP) or sarcoidosis and were excluded [2], [32], [33].

The control subjects were healthy individuals who were undergoing a routine physical examination at the same hospital. Subjects suffering from diagnosed diseases, including acute inflammation, tuberculosis, autoimmune diseases, diabetes and cancers, were excluded from the control group. Moreover, we recruited autoimmune disease patients, including patients with Sjögren's syndrome (SS), rheumatoid arthritis (RA), systemic lupus erythematosus (SLE) and polymyositis/dermatomyositis (PM/DM), as disease controls who were also attending the clinics of the same hospital and were diagnosed by rheumatologists [29], [34]–[36] (Table 1, Table S1).

**Table 1:** Demographic characteristics of the study subjects.

Groups*	No.	Sex (male/female)	Mean age, years
**Controls(all)**	2034	1123/911	40.07±24.64
**Controls(matched)**	1013	525/488	58.61±12.72
**ILD**	405	210/195	59.73±13.1
** IPF**	165	101/64	61.78±12.72
** CTD-ILD**	240	109/131	58.45±13.26
**Pneumonias**	103	65/38	64.20±18.41
**ADs**	360	100/260	40.98±17.79

* ILD = Interstitial lung diseases;

IPF = idiopathic pulmonary fibrosis; CTD = connective tissue diseases; ADs =  autoimmune diseases.

### Specimen collection and DNA extraction

Peripheral venous blood samples (2 ml) were collected in EDTA tubes. The total genomic DNA was extracted from the peripheral blood cells of all index patients and control subjects using a TIANamp Blood DNA Kit according to the manufacturer's instructions (Tiangen BIOTECHCO., Ltd., China).

### PCR amplification

Primers to amplify the *MUC5B* gene (rs35705950) were designed using the Primer Premier 5.0 software. The upstream primer sequence was 5′-GCCCATCCCTGCTTTGTG-3′, and the downstream primer sequence was 5′-CACCTCTGCATCAGCGAGATAG-3′. PCR was performed in a 25-µl reaction mixture containing 1.5 µl of the upstream and downstream primers, 2.0 µl of template DNA, 2.5 µl of MgCl_2_, 1.2 µl of 2.5 mM dNTPs, 2.5 µl of 10 x Taq buffer with (NH_4_)_2_SO_4_, and 0.2 µl of Taq DNA polymerase. The PCR conditions included pre-denaturation for 5 min at 94°C, denaturation for 30 s at 94°C, annealing for 30 s at 58°C, and extension for 30 s at 72°C. The last three steps were repeated for 35 cycles, followed by a final extension for 5 min at 72°C. The PCR yielded an amplicon of 976 bp.

### Variation screening of the *MUC5B* gene by restriction fragment length polymorphism (RFLP)

Fifteen microliters of the PCR product was digested in a reaction mixture that contained 1 µl of the restriction enzyme HaeII (TaKaRa Biotechnology Co., Ltd., Dalian, China), 2 µl of 10× digestion buffer, and 2 µl water for a final volume of 20 µl. The reaction was carried out in a water bath for 16 h at 37°C. Twenty microliters of the digested product was mixed with 2 µl of bromophenol blue and xylene cyanide, and the product was electrophoretically separated on a 1% agarose gel (Invitrogen, Carlsbad, CA) containing ethidium bromide (30 min at 120 V). The gels were observed under UV illumination. The presence of the GG genotype yielded two bands of 616 and 360 bp, and the GT genotype resulted in the appearance of three bands of 976, 616, and 360 bp (Fig. S1).

### Taqman probe-based SNP genotyping for the *MUC5B* variation

Real-time PCR with the TaqMan assay (Applied Biosystems, Foster City, CA) was used to test the reproducibility of the genotyping by RFLP on DNA samples from the patients and healthy controls. This assay was performed according to the manufacturer's instructions. Briefly, 5-µl reactions containing 2.5 µl of the TaqMan universal genotyping master mix, 0.25 µl of TaqMan 20× SNP assay, 0.75 µl of autoclaved reverse osmosis water, and 1.5 µl of DNA (5 ng/µl) per reaction were run. Each run included non-template controls (NTCs). The real-time PCR reactions were performed using a 7300 Fast Real-Time PCR system (Applied Biosystems).

### DNA sequencing

DNA sequencing was performed to confirm the genotype of the polymorphism sites in all heterozygotes and 32 randomly selected homozygous wild-types detected by RFLP and TaqMan assay. The purified PCR products were directly sequenced using an ABI BigDye Terminator v3.1 Cycle Sequencing Kit. The analyses were completed on a 3130 Genetic Analyzer (Applied Biosystems).

### Statistical analysis

The statistical analyses of the cohort study were carried out using the statistical program SPSS version 19.0. The data are expressed as the mean ± SD (continuous variables) and as percent totals (categorical variables). Chi-squared tests with 2×2 contingency tables were used to compare the genotype frequencies in a case–control study, and Student's t test was used to determine differences in the means. The risk for ILD was estimated using an unconditional logistic regression to calculate the odds ratios (OR) and 95% confidence intervals (95% CI). In all cases, p values <0.05 were considered statistically significant.

## Results

### The minor-allele frequency of the rs35705950 was considerably lower in the Chinese Population and significantly differed between Chinese males and females

The demographic characteristics in terms of the sex and age of the subjects are summarized in Table 1. The genotypic frequencies for rs35705950 showed no evidence of a departure from Hardy-Weinberg equilibrium in the control populations. In this work, we screened for this variant in the promoter region of *MUC5B* with RFLP, and a Taqman SNP genotyping assay was used to confirm the genotypes of the investigated subjects. BigDye terminators were used to sequence the PCR products from all detected heterozygous and some of the homozygous subjects (Fig. S1). Our results showed that the allele frequency for the minor-allele of the polymorphism rs35705950 was 0.66% in the healthy controls. The minor-allele frequencies were 2.22% in subjects with ILD, 3.33% in subjects with idiopathic pulmonary fibrosis, 0.7% in subjects with ADs, and 0.49% in subjects with pneumonia (Table 2). The genotyping results for *MUC5B* rs35705950 with the three assays were consistent for all investigated subjects in this study.

**Table 2:** Association of the *MUC5B* rs35705950 polymorphism with ILD, IPF in the Chinese population.

Group*	GG(%)	GT(%)	G(%)	T(%)	p-value†	OR (95% CI)
**Controls** n = 1013	997(98.42)	16(1.58)	2010(99.21)	16(0.79)		
**ILD** n = 405	387(95.56)	18(4.44)	792(97.78)	18(2.22)	**0.002**	**2.855(1.449–5.627)**
**IPF** n = 165	154(93.33)	11(6.67)	319(96.67)	11 (3.33)	**0.001**	**4.332(1.992–9.419)**
**CTD-ILD** n = 240	233(97.08)	7(2.92)	473(98.54)	7(1.46)	0.182	1.859(0.761–4.545)
**CTD-NSIP** n = 191	185(96.86)	6(3.14)	376(98.43)	6(1.57)	0.144	2.005(0.779–5.156)
**CTD-UIP** n = 49	48(97.96)	1(2.04)	97(98.98)	1(1.02)	0.531	1.380(0.181–10.524)
**Pneumonias** n = 103	102(99.03)	1(0.97)	205(99.51)	1(0.49)	1.000	0.613(0.081–4.644)
**Ads** n = 360	355(98.61)	5(1.39)	715(99.3)	5 (0.7)	0.801	0.878(0.321–2.407)

† Determined by χ^2^ test and Fisher's exact test.

OR  =  odds ratio, CI  =  confidence interval.

ILD  =  interstitial lung disease; IPF  =  idiopathic pulmonary fibrosis; CTD  =  connective tissue diseases; NSIP  =  nonspecific interstitial pneumonia pattern; UIP  =  usual interstitial pneumonia pattern; ADs  =  autoimmune diseases.

**Figure 1:**
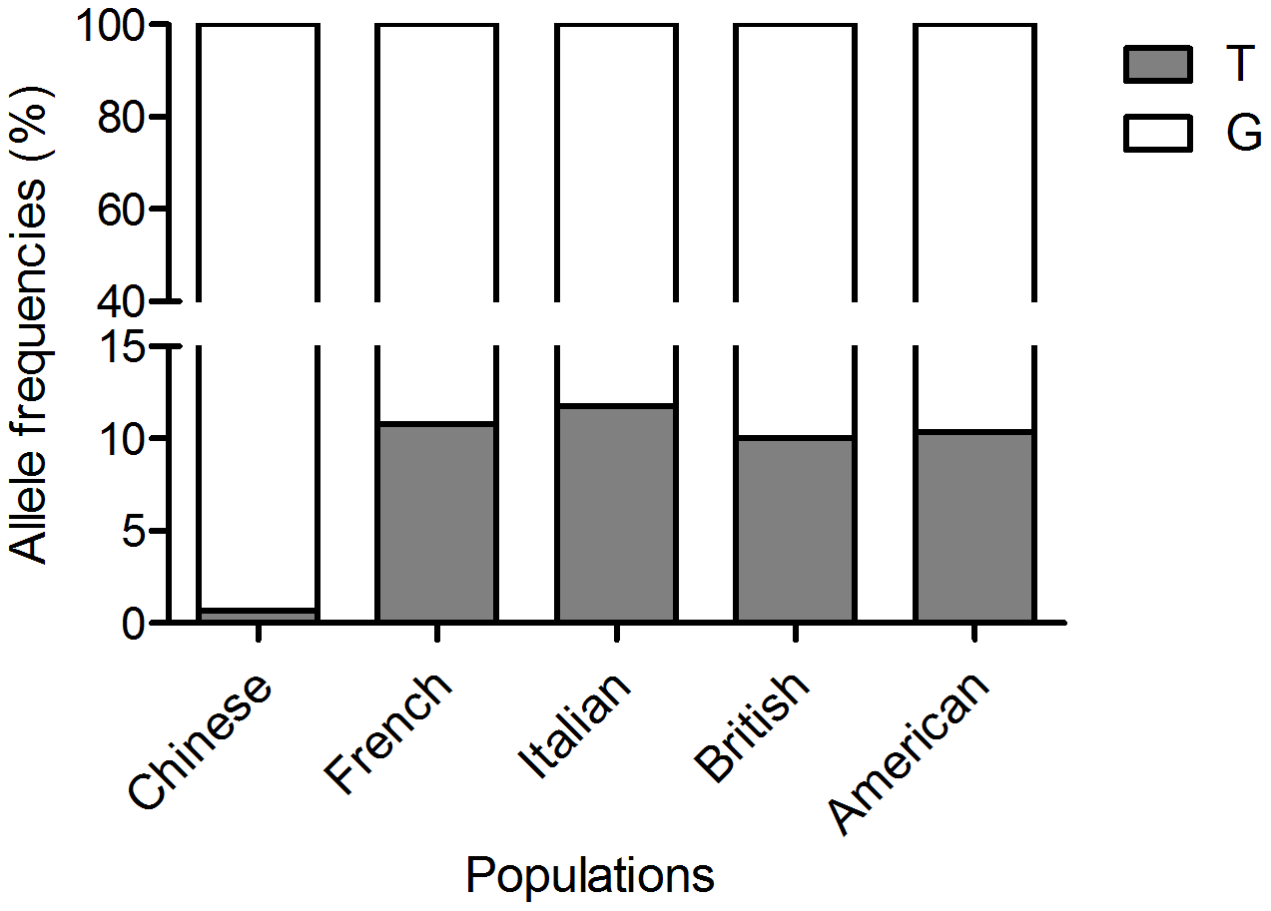
The minor-allele frequency of the rs35705950 in the Chinese population is different from the Caucasian population. Distribution of the *MUC5B* genotype among the Chinese and Caucasian cohorts. Percentage of the G allele is shown in white, and the percentage of the T allele is shown in grey. The data of T allele frequency in Caucasian populations, including Italian, British, French, and American populations, from the references [14], [17]–[20].

Several reports showed that the minor-allele frequency of rs35705950 SNP was approximately 10% among healthy Caucasians in different regions. This variant frequency was only 0.66% in the healthy Chinese population, which was significantly decreased compared with the Caucasian populations. The reported T allele frequencies were similar in the French, American, and English cohorts as well as the Italian IPF population and controls (Fig. 1).

In the healthy Chinese groups, we found that the distribution of the minor-allele (T) differed by gender; the T allele frequency in adult males (≥30 years old) was lower than that in adult females (0.42% versus 1.04%, *P* = 0.030), and no difference in the T allele frequency was detected between the young male and female groups (<30 years old) (Table 3).

**Table 3:** The minor-allele frequency of the *MUC5B* rs35705950 distribution in the normal population.

Group	No.	G*	T*	*P* †
**Female** <30 y	335	666(99.4%)	4 (0.6%)	
**Male** <30 y	403	800(99.26%)	6(0.74%)	1.000
**Female** ≥30 y	576	1140(98.86%)	12 (1.04%)	
**Male** ≥30 y	720	1435(99.58%)	5 (0.42%)	**0.030**

* Allele frequencies were given as No. (%). G: G allele (%); T: T allele (%).

† Determined by χ^2^ test and Fisher's exact t test.

y  =  years.

### Case-control analysis shows an association of the T allele of rs35705950 with ILD and IPF in the Chinese population

We investigated whether the *MUC5B* polymorphism increased the risk for ILD, especially in the Chinese IPF population. The case-control analysis showed a significant association of the rs35705950 SNP with ILD and IPF in the Chinese population (Table 2). The T-allele frequency was 2.22% in ILD patients, which was significantly higher than that in the healthy counterparts (0.79%, *P* = 0.002, OR = 2.885, 95% CI, 1.449–5.627). This minor-allele frequency was 3.33% in IPF patients (*P* = 0.001, OR = 4.332, 95% CI: 1.992–9.419).

### No association was detected between the T allele of rs35705950 and autoimmune connective tissue disease-associated ILD

We found no association between the *MUC5B* variant and the risk for CTD-ILD in both CTD-NSIP (*P* = 0.144) and CTD-UIP (*P* = 0.531). This finding was similar to previous reports regarding systemic sclerosis-ILD (*P* = 0.24) in Caucasian cohorts in the UK [20]. The *MUC5B* polymorphism was not associated with the risk for autoimmune diseases (*P* = 0.801) or pneumonia (*P* = 1.000) in the Chinese population (Table 2).

### Stratified analysis indicated the prevalence of the T allele of rs35705950mainly in the old male ILD patients

In this study, we found that the T allele frequency of the rs35705950 SNP in the healthy male group was lower than that in the healthy female group (0.42% versus 1.04%, *P* = 0.030) (Table 3). We investigated whether age or gender affected the risk for IPF as a function of this polymorphism of the *MUC5B* gene in the Chinese population. A stratified analysis of sex and age indicated that the T allele frequency in the *MUC5B* gene was significantly higher in older (≥55 years) male ILD patients than in the healthy counterparts (3.1% versus 0.37%, *P* = 0.001, OR = 8.152, 95% CI: 2.192–30.314) (Table 4). A significantly higher T allele frequency was found in male IPF patients compared with the corresponding healthy controls (3.75% versus 0.37%, *P* = 0.001, OR = 10.481, 95% CI: 2.593–42.355) (Table 5).

**Table 4:** Stratified analysis of the T allele of *MUC5B* rs35705950 with sex and age in ILD patients.

Sex	Group	No.	G*	T*	*P* †	OR (95% CI)
**Female**<55 y	Controls	185	367(99.19%)	3(0.81%)		
	ILD	75	146 (97.33%)	4(2.67%)	0.110	3.352(0.741–15.159)
**Female**≥55 y	Controls	274	540 (98.54%)	8(1.46%)		
	ILD	120	236 (98.33%)	4(1.67%)	0.762	1.144(0.341–3.836)
**Male** <55 y	Controls	149	296(99.33%)	2(0.67%)		
	ILD	57	113 (99.12%)	1(0.88%)	1.000	1.310(0.118–14.586)
**Male** ≥55 y	Controls	405	807(99.63%)	3(0.37%)		
	ILD	153	297(97.06%)	9 (2.94%)	**0.001**	**8.152(2.192–30.314)**

* Allele frequencies were given as No. (%). G: G allele (%); T: T allele (%).

† Determined by χ^2^ test and Fisher's exact t test.

OR  =  odds ratio, CI  =  confidence interval, y  =  years. ILD  =  interstitial lung disease.

**Table 5:** Stratified analysis of the T allele of *MUC5B* rs35705950 with sex and age in IPF patients.

Sex	Group	No.	G*	T*	*P* †	OR (95% CI)
**Female**<55 y	Controls	185	367(99.19%)	3(0.81%)		
	IPF	23	44 (95.7%)	2 (4.3%)	0.096	5.561(0.904–34.193)
**Female**≥55 y	Controls	274	540 (98.54%)	8(1.46%)		
	IPF	39	75 (96.15%)	3(3.85%)	0.147	2.700(0.701–10.402)
**Male** <55 y	Controls	149	296(99.33%)	2(0.67%)		
	IPF	21	42 (100%)	0 (0%)	1.000	1.007(0.997–1.016)
**Male** ≥55 y	Controls	405	807(99.63%)	3(0.37%)		
	IPF	80	154(96.25%)	6(3.75%)	**0.001**	**10.481(2.593–42.355)**

* Allele frequencies were given as No. (%). G: G allele (%); T: T allele (%).

† Determined by χ^2^ test and Fisher's exact t test.

OR  =  odds ratio, CI  =  confidence interval, y  =  years. IPF  =  idiopathic pulmonary fibrosis.

## Discussion

In this study, we investigated the relationship between the rs35705950 polymorphism of the *MUC5B* gene and the risk for ILD in Chinese populations. We first screened this variant with RFLP. The minor-allele (T) of this polymorphism was detected in only 27 individuals as the heterozygous genotype, and no homozygous T/T genotype was found among the 2034 healthy Chinese individuals, indicating that the T-allele frequency of the rs35705950 polymorphism was approximately 0.66% in the Chinese population. The T-allele frequency was reported to be 10.81% in the French population, 11.74% in the Italian population, 10% in the United Kingdom population, and 9.1% in the USA-Denver Caucasian population. This risk T allele frequency of the rs35705950 polymorphism was less than one-tenth in the Chinese population compared with that in the Caucasian populations. We used the TaqMan probe and direct sequencing techniques ensure the reproducibility of the genotypes for the polymorphism sites of the investigated subjects. The consistency of these results obtained with three methods indicated that the prevalence of the rs35705950 variant appears to be much lower in the Chinese population than in the Caucasian population. The published data of the 1000 Genomes Project showed only one heterozygote of the *MUC5B* rs35705950 detected in 100 Chinese subjects.

However, our results supported a strong association between the T-allele of the *MUC5B* rs35705950 and the risk for ILD, especially for IPF, in the Chinese population, which is similar to the association described in the Caucasian population in Europe and the USA [14], [18], [20]. The low prevalence of the *MUC5B* variant rs35705950 suggests that it might not be the main risk factor for idiopathic pulmonary fibrosis in the Chinese population. We screened for the variant of rs17235353, a 2-bp deletion/insertion polymorphism in the putative promoter of *MUC5B*, which had been described to associate with diffuse panbronchiolitis in Asians [37]. However, no association was found between the variant of *MUC5B* rs17235353 and the IPF risk (data not shown).

Lung involvement, particularly interstitial lung disease (ILD), could be the first manifestation of autoimmune connective tissue diseases (CTDs). ILD could occur in all CTDs, although its prevalence in these diseases varied [25]. In IPF, disease progression is almost invariable, while CTD-ILD is manifested by a variable rate of progression, with a substantial proportion of patients showing limited and stable disease [38]. Several studies reported that the T allele of *MUC5B* rs35705950 was associated with idiopathic pulmonary fibrosis but not with the development of lung fibrosis in systemic sclerosis or sarcoidosis [18]-[20]. In this study, we focused on the most frequent autoimmune CTDs, including SS, RA, SLE, and PM/DM, and investigated the prevalence of the T-allele of the *MUC5B* rs35705950 in CTD-ILD patients. Our results showed that this putative promoter variant of *MUC5B* was not associated with CTD-ILD, neither for CTD-NSIP nor for CTD-UIP. The lack of an association with lung fibrosis in the context of autoimmune connective tissue diseases suggests that this *MUC5B* variant could not be related to share fibrotic mechanisms across CTD-ILD.

MUC5B is the major gel-forming mucin in the normal distal airway epithelium [14], [39]. The aberrant high expression of MUC5B has been observed in the lungs of patients with IPF and may be related to the abnormal differentiation of the respiratory epithelium [40]. Seibold et al. reported that *MUC5B* mRNA expression increased 14.1-fold in the lung tissues of IPF patients compared with that in unaffected subjects. The expression level of *MUC5B* mRNA in the lung tissues of unaffected subjects carrying the T-allele of *MUC5B* rs35705950 was much higher than that of unaffected subjects homozygous for the wild-type allele [14]. Borie et al. reported that the distribution of the rs35705950 T-allele risk did not differ by gender in Caucasian populations [20]. They considered that the rs35705950 variant could have an independent effect on the susceptibility to IPF. In addition, smoking often appeared as a confounder for increasing MUC5B expression. Long-term cigarette smoking could increase the level of MUC5B expressed by lung alveolar macrophages. Smoking also showed a dose-related association with an increased risk for IPF [41]–[45].

Interestingly, our results indicated that the frequency of the T-allele of *MUC5B* rs35705950 in healthy adult male individuals (≥30 yrs) was lower than in matched female individuals (*P* = 0.030). The rs35705950 T allele was more prevalent in older male patients (≥55 yrs) with ILD or IPF than in patients less than 55 years old. The prevalence of the T allele of *MUC5B* could have been significantly lower in healthy males, especially in the older group, because those males carrying the T allele of *MUC5B* were excluded from the healthy population due to illness. Moreover, we failed to detect any significant difference in the T-allele frequencies between female ILD patients and healthy female controls as well as between female IPF patients and matched controls. The epidemiological data showed that the smoking rates were high among both males and females in Europe. The proportion of smokers among Italian males was approximately 30.2% and 18.8% among females in 2009 [46]. In France, the male smoking rate was approximately 36%, and the female smoking rate was 27% in 2012 [47]. According to the 1996 national prevalence survey in China, approximately two-thirds of Chinese men were smokers, but less than 4% of women had ever smoked [48]. A 2010 survey by the World Health Organization (WHO) revealed that more than half of Chinese men were still smoking, but less than 3% of women smoked. Among the patients recruited in this study, 58% of the male IPF patients had been smokers, but none of the women IPF patients had ever smoked (data not showed). The low smoking rates among Chinese women might be one of the important reasons for the lack of association detected between the T allele of *MUC5B* and female IPF in this study. We hypothesized that smoking exposure could promote the pathogenesis of IPF in subjects with the *MUC5B* rs35705950 variant. Further studies are required to define the association between the *MUC5B* variant and the smoking exposure in the pathogenesis of IPF.

Our result demonstrated that the minor-allele frequency of the polymorphism rs35705950 in the Chinese populations is lower than that in Caucasians populations. This variant significantly associated with the risk for ILD/IPF in Chinese subjects, although it might not be the main risk factor for IPF in the Chinese population. Because the number of ILD patients recruited was limited in this study, especially for the stratified analysis by sex and age, further study will be required to define the prevalence of the *MUC5B* rs35705950 variant in the Chinese population.

## Supporting Information

Figure S1 **Variation screening and confirmation the rs35705950 SNP in the promoter region of the** ***MUC5B*** **gene.** (A) PCR-RFLP analysis of the genotypes. Lane 1: M marker; Lanes 3, 4, 6, 7, 9, 10: the G/T heterozygote genotype was visualized as bands of 976+616+360 bp; Lanes 2, 5, 8: the G/G homozygote genotype as visualized as bands of 616+360 bp. (B) Direct sequencing of the PCR products indicated a heterozygous G/T mutation and a homozygous G/G mutation. (C) TaqMan SNP genotyping of the indicated promoter polymorphisms in the *MUC5B* gene. G/T (green dots), and G/G (red dots), no detection of T/T (blue dots). The black dots in the bottom-left are the negative control, the black forks are the undetermined samples, and all of the undetermined samples were re-tested.
(TIF)

Table S1 **The demographic characteristics of autoimmune diseases.**
(DOCX)
