# Supplementary figures and images for: Mucin 5B Promoter Polymorphism Is Associated with Susceptibility to Interstitial Lung Diseases in Chinese Males
Source: PLoS One. 2014 Aug 14;9(8):e104919. doi: 10.1371/journal.pone.0104919 (PMC4133265; doi:10.1371/journal.pone.0104919)

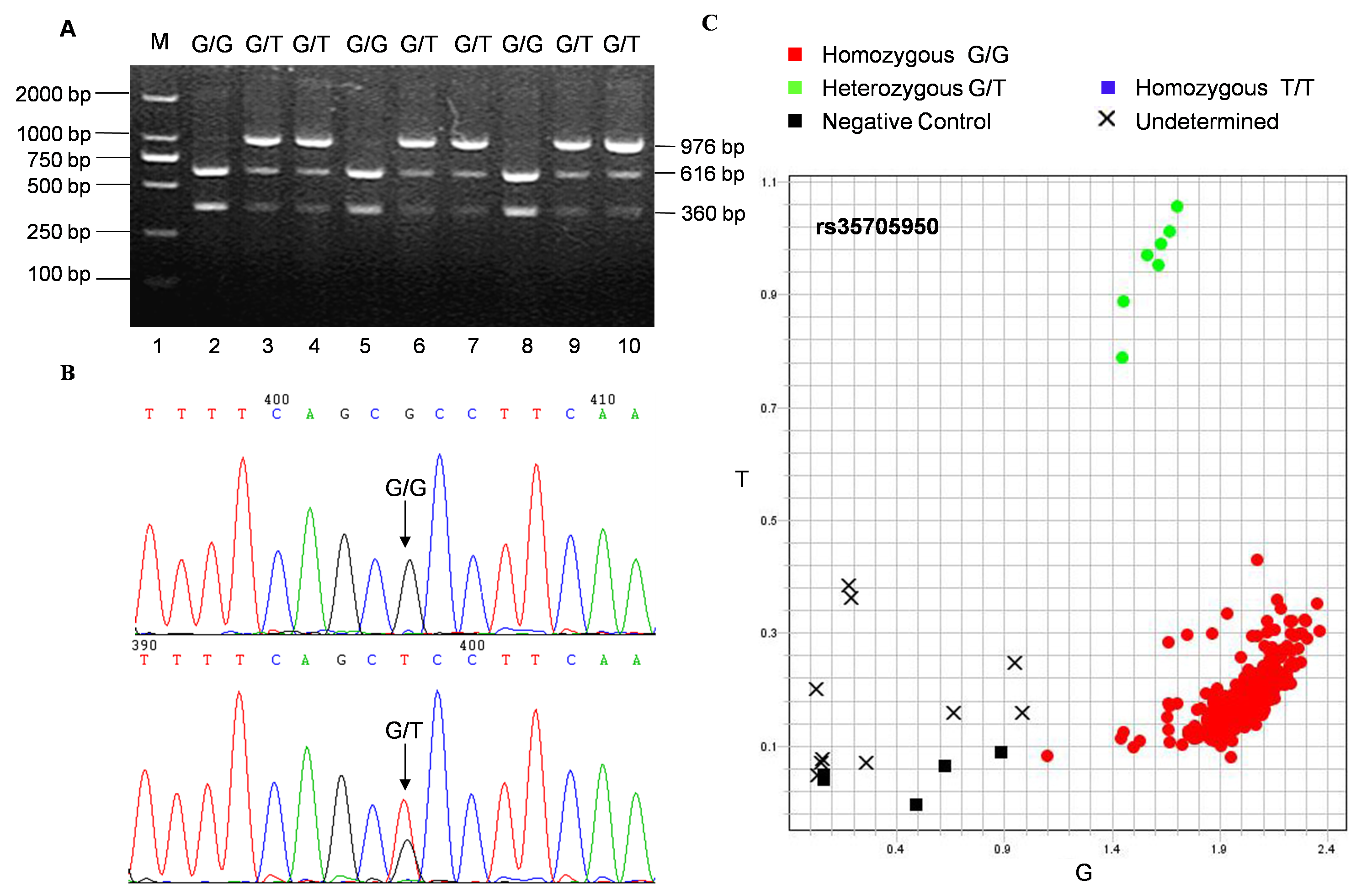

Supplement: Figure S1 — Variation screening and confirmation the rs35705950 SNP in the promoter region of the MUC5B gene. (A) PCR-RFLP analysis of the genotypes. Lane 1: M marker; Lanes 3, 4, 6, 7, 9, 10: the G/T heterozygote genotype was visualized as bands of 976+616+360 bp; Lanes 2, 5, 8: the G/G homozygote genotype as visualized as bands of 616+360 bp. (B) Direct sequencing of the PCR products indicated a heterozygous G/T mutation and a homozygous G/G mutation. (C) TaqMan SNP genotyping of the indicated promoter polymorphisms in the MUC5B gene. G/T (green dots), and G/G (red dots), no detection of T/T (blue dots). The black dots in the bottom-left are the negative control, the black forks are the undetermined samples, and all of the undetermined samples were re-tested. (TIF) [file pone.0104919.s001.tif]
