# Supplementary material for: Mucin 5B Promoter Polymorphism Is Associated with Susceptibility to Interstitial Lung Diseases in Chinese Males
Source: PLoS One. 2014 Aug 14;9(8):e104919. doi: 10.1371/journal.pone.0104919 (PMC4133265; doi:10.1371/journal.pone.0104919)
Supplement: Table S1 — The demographic characteristics of autoimmune diseases. (DOCX) [file pone.0104919.s002.docx]

**Table S1.** The demographic characteristics of autoimmune diseases.

| **Groups*** | **No.** | **Sex (male/female)** | **Mean age, years** |
| --- | --- | --- | --- |
| Sjögren’s syndrome | 73 | 5/70 | 45.15±13.06 |
| Rheumatoid arthritis | 56 | 12/44 | 54.06±12.85 |
| Systemic lupus erythematosus | 116 | 10/106 | 34.52±11.93 |
| Poly-/dermatomyositis | 33 | 9/24 | 42.17±18.09 |
| Other autoimmune diseases | 82 | 64/18 | 41.82±19.67 |
